# Development of a clinical tool for rating the body function categories of the ICF generic-30/rehabilitation set in Japanese rehabilitation practice and examination of its interrater reliability

**DOI:** 10.1186/s12874-021-01302-0

**Published:** 2021-06-14

**Authors:** Yuki Senju, Masahiko Mukaino, Birgit Prodinger, Melissa Selb, Yuki Okouchi, Kouji Mizutani, Megumi Suzuki, Shin Yamada, Shin-Ichi Izumi, Shigeru Sonoda, Yohei Otaka, Eiichi Saitoh, Gerold Stucki

**Affiliations:** 1 Department of Rehabilitation Medicine I, School of Medicine, Fujita Health University, 1-98 Dengakugakubo, Kutsukake, Toyoake, Aichi 470-1192 Japan; 2 Faculty of Applied Health and Social Sciences, Technical University of Applied Sciences Rosenheim, Rosenheim, Germany; 3 Swiss Paraplegic Research, Nottwil, Switzerland; 4 ICF Research Branch, Nottwil, Switzerland; 5 Department of Health Sciences and Medicine, University of Lucerne, Lucerne, Switzerland; 6 Department of Rehabilitation, Fujita Health University Hospital, Toyoake, Aichi Japan; 7 Faculty of Rehabilitation, School of Health Sciences, Fujita Health University, Toyoake, Aichi Japan; 8 Department of Rehabilitation Medicine, Kyorin University School of Medicine, Tokyo, Japan; 9 Department of Physical Medicine and Rehabilitation, Tohoku University Graduate School of Medicine, Sendai, Miyagi Japan; 10 Department of Rehabilitation Medicine II, School of Medicine, Fujita Health University, Tsu, Mie Japan

**Keywords:** Rehabilitation, International Classification of Functioning, Disability, and Health, Interrater reliability, Clinical tool

## Abstract

**Background:**

The International Classification of Functioning, Disability, and Health (ICF) Generic-30 (Rehabilitation) Set is a tool used to assess the functioning of a clinical population in rehabilitation. The ICF Generic-30 consists of nine ICF categories from the component “body functions” and 21 from the component “activities and participation”. This study aimed to develop a rating reference guide for the nine body function categories of the ICF Generic-30 Set using a predefined, structured process and to examine the interrater reliability of the ratings using the rating reference guide.

**Methods:**

The development of the first version of the rating reference guide involved the following steps: (1) a trial of rating patients by several raters; (2) cognitive interviews with each rater to analyze the thought process involved in each rating; (3) the drafting of the rating reference guide by a multidisciplinary panel; and (4) a review by ICF specialists to confirm consistency with the ICF. Subsequently, we conducted a first field test to gain insight into the use of the guide in practice. The reference guide was modified based on the raters’ feedback in the field test, and an inter-rater reliability test was conducted thereafter. Interrater agreement was evaluated using weighted kappa statistics with linear weights.

**Results:**

The first version of the rating reference guide was successfully developed and tested. The weighted kappa coefficient in the field testing ranged from 0.25 to 0.92. The interrater reliability testing of the rating reference guide modified based on the field test results yielded an improved weighted kappa coefficient ranging from 0.53 to 0.78. Relative improvements in the weighted kappa coefficients were observed in seven out of the nine categories. Consequently, seven out of nine categories were found to have a weighted kappa coefficient of 0.61 or higher.

**Conclusions:**

In this study, we developed and modified a rating reference guide for the body function categories of the ICF Generic-30 Set. The interrater reliability test using the final version of the rating reference guide showed moderate to substantial interrater agreement, which encouraged the use of the ICF in rehabilitation practice.

## Background

The International Classification of Functioning, Disability, and Health (ICF) is a framework for describing and organizing information on functioning and disability [1, 2]. Since the ICF was endorsed in May 2001, various initiatives have been undertaken to promote its implementation [3–8], including the development of ICF core sets based on a multi-modal international and interprofessional process. ICF core sets contain selected categories from the entire classification that can serve as minimum standards for assessing and documenting the functioning and health of individuals with a specific disease or disorder. In addition, two ICF sets were developed for generic use. The ICF generic set (also called ICF Generic-7 Set) consists of seven ICF categories that are considered most relevant for assessing and documenting the functioning of the general population as well as different clinical populations irrespective of health condition, contexts, settings, and purposes [9]. The ICF rehabilitation set (also called ICF Generic-30 Set) is an extended version of the ICF Generic-7 Set comprising 30 ICF categories, and is used in the context of rehabilitation and disability to describe varying levels of functioning across various clinical populations and along the continuum of care [10, 11]. Although the ICF core sets provide a specification of which domains to assess, they do not stipulate how to assess them.

In the ICF, the World Health Organization (WHO) proposed a rating system that consists of so-called "qualifiers" that can be used to code the severity of functioning problems. The qualifiers are as follows: 0, no problem; 1, mild problem; 2, moderate problem; 3, severe problem; 4, complete problem; 8, not specified; and 9, not applicable [1]. Unlike most existing clinical scales, there are no detailed or additional explanations for using qualifiers for rating functioning. The lack of more detailed guidance on how to use qualifiers may make rating of problems patients experience in a given ICF category more difficult, potentially leading to inconsistent ratings. For example, Uhlig et al. examined the interrater reliability of clinician ratings using ICF qualifiers and the ICF core set for rheumatoid arthritis and reported low reliability [12]. The low reliability of these ratings is particularly problematic in promoting clinical implementation of the rating scale. In objective clinical assessment using clinical scores, it is important to produce consistent results across raters and over time [13]. Since the assessment of functioning is usually performed by multidisciplinary professionals and used to exchange patient information, it is particularly important to ensure that the scores have the same meaning among the various raters. To achieve this, in addition to the existing simple guidelines, the development of more concrete complementary explanations would be helpful. To identify the potential for improving the interrater reliability of clinician ratings using ICF core sets, Mukaino et al. conducted a multistage study using the activities and participation categories of the ICF Generic-30 Set [14]. Specifically, a rating guide for the activities and participation categories was developed and modified based on the results of a cognitive interview of clinicians who field tested the guide. The rating guide employed the 0–4 qualifier rating scale, as this had been shown to perform well in another study when used in the activities and participation component [15]. The interrater reliability using this modified version of the rating guide was moderate to substantial. However, developing such a rating reference guide for body function categories may be more difficult. While problems in activities and participation can be rated relatively easily by indicating, for example, whether a person is able, conditionally able, or not able to perform a particular activity, as is done with existing clinical rating scales [16, 17], body function categories cannot be explained by a single factor (e.g., able to perform). Multiple factors must be considered to determine the magnitude of the body function problem. For example, the problems in category b280 sensation of pain have several aspects, such as the extent of pain, pain frequency, or the site of pain. Thus, the rating could vary depending on the aspect the rater focuses on. One clinician may focus on the frequency of pain, while another may focus on the maximum pain experienced by the patient. Furthermore, one may only ask patients about the intensity of the pain, while another may only consider the site of the pain. Thus, specifying what the category is addressing, for example, in the form of a guidance document with category specifications, can help clinicians make an informed judgment for rating.

In this study, we aimed to create a rating reference guide for the nine body function categories of the ICF Generic-30 Set, which leads to reliable ratings.

## Materials and methods

**Fig. 1:**
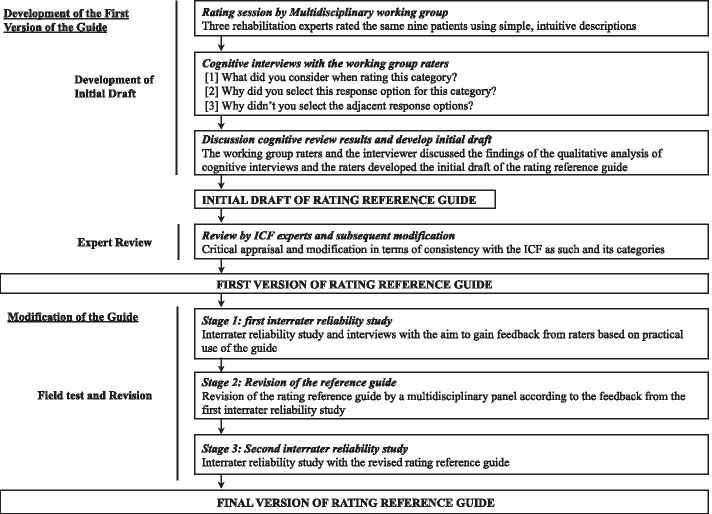
Flow diagram for the development process of the rating reference guide

The development and assessment of the rating reference guide were conducted according to the flowchart shown in Fig. 1.

### Development of the first version of the rating reference guide

Prior to the development of the rating reference guide, the thought processes of clinicians when rating using qualifiers were assessed through a cognitive interview. In this study, a multidisciplinary working group with ICF research experience was organized to develop the initial draft of the reference guide. The group consisted of a physiatrist, a physical therapist, and an occupational therapist from the same hospital. The members of the working group independently evaluated the functioning of the same nine patients (three acute patients, three subacute patients, and three chronic patients; age, 56.7 ± 18.6; seven males and two females; five with neurological diseases, three with orthopedic diseases, and one with respiratory disease) in the hospital using the ICF Generic-30 Set and the original qualifier scoring [1]. The terms “acute”, “subacute”, and “chronic” mentioned above indicate time periods within 14 days, 15 to 120 days, and more than 120 days after onset of the disease, respectively. The Japanese version of the simple, intuitive descriptions of the ICF Generic-30 Set [14] was used to facilitate the understanding of the category, which also supported the determination of the ratings. The Japanese-language simple, intuitive descriptions of the ICF Generic-30 Set were developed based on an established consensus process that has been promoted by national physical and rehabilitation medicine societies worldwide [11, 18, 19]. Cognitive interviews were conducted after the evaluation was completed, during which working group members who rated the patients were asked the following questions:

1. What did you consider when rating this category?
2. Why did you select this response option for this category (for example, why did you select qualifier 2 for the category “d450 walking”)?
3. Why did you not select the adjacent response options (for example, why did you select qualifier 2 instead of 3?

The interview was conducted by a physiatrist researcher with 15 years of experience in rehabilitation clinics and ICF research. Subsequently, a qualitative content analysis of the cognitive interviews was conducted. For content analysis, inductive content analysis procedures were used [20]. First, the text of the raters was grouped into the following categories: 1) the factors to consider in the rating and 2) the reasons for selecting or not selecting each response. The open coding and organization of the codes into categories and themes for each group was performed by the researcher who conducted the interviews. The results of the analysis were then examined and discussed with another researcher who was not involved in the rating session. This was accomplished to check the accuracy and appropriateness of the coding and categorization. Using the results of these analyses, the working group raters were asked to discuss and develop a simple rating reference guide. The researcher who conducted the individual interviews and conducted content analysis moderated the discussion and guided the development process. The working group raters were asked to develop a rating reference guide for a rating scale of 0 to 4, to keep the guide as simple as possible, and to keep the response options of the scale consistent across the categories. The ICF qualifier response options “8: not specified” and “9: not applicable” were maintained in order to be consistent with the structure of the ICF qualifiers. The draft guide was then reviewed and modified by a multidisciplinary group of eight ICF experts with regard to consistency and simplicity. The resulting document was regarded as the first version of the rating reference guide for the body function categories of the ICF Generic-30 Set with a 0–4 rating scale (from now on referred to as “first version of the rating reference guide”).

### Modification of the guide—stage 1: first interrater reliability study

A preliminary interrater reliability study using the first version of the rating reference guide was performed to obtain feedback on the use of the guide in real-life clinical practice. The guide was field-tested by four independent raters (two physiatrists, one physical therapist, and one occupational therapist). The raters consisted of two clinicians who participated in the developmental process of the guide. Two clinicians that served as raters were also randomly selected from middle managers in the rehabilitation department of the hospital. Each patient involved in the field test was rated by two of the four raters.

After the raters completed patient evaluations using the rating reference guide, a researcher interviewed the four raters to determine what the raters found difficult in rating with the first version of the rating reference guide. The raters were asked the following questions:

1. Did you have difficulty in rating with this guide?
2. If yes, what made it difficult for you to rate?

A qualitative analysis on the results of the interview was then conducted.

### Modification of the guide—stage 2: revision of the reference guide

After the field test, a multidisciplinary panel consisting of two physiatrists, two physical therapists, and two occupational therapists, was organized to modify the first version of the rating reference guide based on the feedback resulting from the field test. Four clinicians from the panel were also involved in the development process. Two clinicians (a physical therapist and an occupational therapist) who had experience with ICF research were new additions to the panel. The panel was asked to discuss ideas to address the issues raised by the raters during the field test. The panel members considered the results of the interview of the four field test raters, as well as the record of the cognitive interview used to develop the initial draft, and discussed how to modify the guide to make it easier for clinicians to assign ratings.

### Modification of the guide—stage 3: second interrater reliability study

A second interrater reliability test was conducted with four raters (two physical therapists and two occupational therapists). The raters were randomly selected from middle managers in the rehabilitation department of the hospital. They were not involved in the first interrater study. Consequently, no rater in the second interrater study was involved in the developmental process or in the first interrater reliability study. Each patient was rated by two of four raters.

The sample size required for a rigorous reliability study was determined by the number of response options (five), the minimum value for the desired kappa coefficient (0.3, for every ICF category), and the power (90.0%) and alpha (0.05). The minimum sample size was 36 [21, 22]. Accordingly, the minimum sample size for each rater was set at 36 patients, excluding missing values. The two pairs of raters each evaluated a different group of 36 patients who had received rehabilitation.

### Data analysis

Weighted kappa statistics were used to determine inter-rater agreement among raters in both interrater reliability studies. Weighted kappa statistics with linear weights[23] were calculated for each item of the body function categories of the ICF Generic-30 Set. Response options 8 (“not specified”) and 9 (“not applicable”) on the qualifier scale were not included in the kappa statistics and were considered as missing data. The standards for interpreting the kappa coefficients were as follows: ≤ 0.20, poor; 0.21–0.40, fair; 0.41–0.60, moderate; 0.61–0.80, substantial; and ≧ 0.81, excellent [24]. The relative improvements in kappa statistics in the second study were calculated with the following formula;

$$\frac{{\kappa }_{2}-{\kappa }_{1}}{{\kappa }_{1}}$$

κ_1_, κ_2_: weighted kappa coefficients in the first study (κ_1_) and the second study (κ_2_).

## Results

### Development of the first version of the rating reference guide

Table 1 shows the first version of the rating reference guide for the body function categories of the ICF Generic-30 Set. In developing the rating reference guide, two key topics emerged from the qualitative content analysis of the discussion notes: what to rate in each category, and how to frame the response options.

**Table 1:** First version of the rating reference guide

		What aspect should be scored		What is the complete problem
b130	Energy and drive functions	・The extent of the problem		May include: Having no motivation or appetite at any time
		・The frequency of the problem		
b134	Sleep functions	・The extent of the problem		May include: Incapable of sleep at all, or the sleeping schedule has collapsed
		・The frequency of the problem		
b152	Emotional functions	・The extent of the problem		May include: Complete loss of control of emotions every day, or incapable of expressing emotions
		・The frequency of the problem		
b280	Sensation of pain	・The extent of the problem		May include: Suffering from continuous intolerable pain at any time
		・The frequency of the problem		
b455	Exercise tolerance functions	・The extent of the problem		May include: Incapable of bearing any single activity of daily living at any time due to cardiorespiratory problems
		・The frequency of the problem		
b620	Urination functions	・The extent of the problem		May include: Complete urinary retention or continuous incontinence at any time
		・The frequency of the problem		
b640	Sexual functions	・The extent of the problem		May include: Having no mental and/or physical ability to perform sexual activity, or complete loss of control in sexual desire at any time
		・The frequency of the problem		
b710	Mobility of joint functions	・The extent of the problem		May include: Complete joint contracture in all major joints
		・The ratio of the joint with the problem		
b730	Muscle power functions	・The extent of the problem		May include: Complete loss of muscle power in all major muscles
		・The ratio of the joint with the problem		
Ratings	(Note that the rating should reflect the body function without the help of devices)			
0	No problem			
1	Mild problem			
2	Moderate problem			
3	Severe problem			
4	Complete problem			
8	Not specified			
9	Not applicable			

#### What to rate in each category

The challenges posed by having several aspects (e.g., severity, frequency, location) to consider in rating a given category were discussed. For example, for the category “b134 sleep functions” the problem could be understood as a combination of the frequency and the extent of problems in sleep functions. To address these challenges, working group members highlighted specific aspects when evaluating each category. The overall sentiment was that having too many aspects to focus on would complicate the rating process and that the rating reference guide should be kept as simple as possible. Accordingly, the working group members identified two main aspects that should be considered when rating the body function categories of the ICF Generic-30 Set – the extent and frequency of the problem in the given category.

#### How to frame the response options

The working group members also proposed that concrete examples should be provided to improve the clarity of the guide, and that these examples should include information on clinical instruments that are commonly used to assess the given category. This idea was first adopted during the development of the initial draft; however, during the review process by ICF experts, there was a concern that this approach may result in overly complicated manuals. Since every category would highlight several aspects that should be considered during rating, the manuals would have to include specific descriptions of those aspects as well as define how to consider a combination of problems. For example, if we rate the status of muscle functions with manual muscle testing, then we should also consider how many and which muscles are impaired. Several reviewers raised concerns that this would make the use of the rating reference guide in the clinic too complicated. Accordingly, concrete descriptions based on the clinical instruments in the reference guide were removed from the initial draft.

The first version of the rating reference guide was simplified as follows: Two major aspects were specified and should be considered when assigning ratings (see Table 1) for each category. For example, for “d620 urination function,” the guide instructs raters to consider the frequency and extent of the problem when rating this category from 0 to 4. No further explanation regarding each response option has been provided.

### Modification of the guide—stage 1: first interrater reliability study

Sixty individuals (52 inpatients and 8 community-dwelling elderly) participated in this study. The 52 patients underwent rehabilitation between April 2017 and November 2018 at Fujita Health University Hospital and Fujita Health University Nanakuri Memorial Hospital. Among these 60 individuals (38 males and 22 females), 31 had neurological disease, 9 had musculoskeletal disease, 9 had cardiopulmonary disease, and 4 had other health issues. The mean age of the patients was 64.5 ± 17.7 years.

The results of the inter-rater reliability calculation of the ratings using the initial version of the rating reference guide are shown in Table 2. The weighted kappa statistics ranged from 0.25 to 0.92, indicating low interrater reliability for several categories, and moderate to excellent for other categories. For example, the weighted kappa coefficients for “b620 urination functions” indicated excellent interrater reliability, while the kappa for “b152 emotional functions” indicated fair interrater reliability [24]. A high rate of missing data (68.3%) was observed for “b640 sexual functions”.

**Table 2:** Interrater reliability of the rating using the first version of the rating reference guide

	Categories	Weighted κ	95% CI	Missing values
b130	Energy and drive functions	0.56	0.35–0.77	0
b134	Sleep functions	0.62	0.45–0.77	0
b152	Emotional functions	0.25	0.01–0.49	0
b280	Sensation of pain	0.44	0.27–0.62	1 (1.7%)
b455	Exercise tolerance functions	0.55	0.39–0.71	0
b620	Urination functions	0.92	0.83–1.01	0
b640	Sexual functions	0.80	0.53–1.07	41 (68.3%)
b710	Mobility of joint functions	0.58	0.41–0.74	0
b730	Muscle power functions	0.65	0.51–0.79	0

*95%CI* 95% confidence interval

According to the results of the first field test, the guidance information for the following four categories with the lowest interrater reliability were identified as needing improvement: “b130 energy and drive functions,” “b152 emotional functions”, “b280 sensation of pain,” and “b455 exercise tolerance functions”. The following problems raised during the field test were discussed in an effort to improve the interrater reliability of these categories: difficulty distinguishing between mild and moderate problems, difficulty rating patients who cannot express their emotions (“b152 emotional functions”), and lack of consideration of the number of pain sites (“b280 sensation of pain”).

Two of the raters indicated that it was difficult to distinguish between mild and moderate problems. The ICF published by the World Health Organization states that a moderate problem is “generally up to half of the scale of the total problem” [1]; thus, the raters felt that differences between moderate and severe problems were relatively easy to distinguish. However, other than this clarifying statement about the interpretation of a moderate problem, there are no clarifications about mild problems, only the presentation of the corresponding percentages of mild and moderate problems (5%–24% and 25%–49%, respectively). This makes it difficult to differentiate between mild and moderate problems. This lack of guidance was problematic when rating patients in the clinic.

The second point raised by the raters was the complexity of the first version of the rating reference guide. The first version of the rating reference guide outlined specifically for each category includes various aspects to be rated and an example of what a complete problem would encompass. The raters were required to take this information into account when rating each category, but without a concrete guide for each response option (0 to 4). Several raters stated that these rating instructions were confusing and made rating difficult.

An issue related to "b152 emotional functions" was also raised. Specifically, a rater highlighted the difficulty in evaluating the emotional functions of patients with problems expressing emotions. The first version of the guide indicated that a “complete problem” in b152 is exemplified by the complete loss of emotion control every day. However, there are patients who do not lose control of emotions, but are unable to express emotions. Three of the raters agreed that the inability to express emotions should also be recognized as a problem in b152.

With regard to rating “b280 sensation of pain” the first version of the rating reference guide instructed that frequency, and the extent of pain should be considered when rating. However, two of the raters indicated that the number of pain sites also influenced the degree of the problem.

### Modification of the guide—stage 2: revision of the reference guide

The reference guide was modified by a multidisciplinary panel according to feedback from the raters in the field test. As the feedback from the raters was focused on the issues in the rating and did not always include concrete suggestions for improvement, the investigators decided to revisit the records of the first cognitive interviews that resulted in the initial draft of the guide (see Fig. 1). This helped to address the difficulty in distinguishing between mild and moderate problems. According to the interview records, several raters mentioned that assigning a rating of 1 (mild problem) for five of the nine categories (“b130 energy and drive functions,” “b134 sleep functions,” “b280 sensation of pain,” “b710 mobility of joint functions,” and “b730 muscle power functions”) was due to the lack of impact these body functions had on daily activities. Given this, the members of the panel added text to the guide that describes a mild problem in a particular ICF category as a problem that does not affect daily activities. To clarify the difference between a rating of 2 (moderate problem) and a rating of 3 (severe problem), the following explanations were given: A rating of 2 “may include a problem that exceeds a rating of 1, but still remains a relatively minor problem (< 50%) in the given category”, and a rating of 3 “may include a problem that is a major problem (≧ 50%) in the given category”. The percentages (< 50% and ≥ 50%) were added to emphasize that a “moderate problem” is “generally up to half of the scale of the total problem” [1]. This further distinguishes ratings 2 and 3. The percentage was set to describe how much the problem was relative to a complete problem (100% as the amount of the problem). For example, in scoring "b130 energy and drive functions", a complete problem is described as “having no motivation nor appetite at any time”, and this is regarded to be 100% of the problem. Raters then consider the amount of problem a person has in b130 by considering the extent and frequency of the lack of motivation or appetite.

The rating reference guide descriptions for “b152 emotional functions” and “b280 sensation of pain” were also modified. For “b152 emotional functions,” the following explanation describing a complete problem in this category was added: “being incapable of expressing emotions at any time” For “b280 sensation of pain,” the pain site was added as an aspect to be considered prior to assigning a rating score. The modified rating reference guide (hereafter referred to as “final version of the rating reference guide”) is shown in Table 3.

**Table 3:** Second version of the rating reference guide

Category	Aspect to be scored	Description of each response option (The percentage describes the severity of the problem, if 100% means it is a complete problem. The rating should reflect the body’s function without the help of devices)	
b130	Energy and drive functions	Extent and frequency of the problem, such as loss of motivation or appetite	0: No problem
			1: Mild problem: May include problems with energy and drive functions that do not affect the patient's daily activities
			2: Moderate problem: May include a problem in energy and drive functions that exceeds 1, but remains a relatively minor problem (< 50%)
			3: Severe problem: May include a major problem (≧ 50%) in energy and drive functions
			4: Complete problem: May include a complete problem with energy and drive functions, such as having no motivation or appetite at any time
b134	Sleep functions	Extent and frequency of the problem, such as shortage of sleep or irregular sleep schedules	0: No problem
			1: Mild problem: May include problems with sleep that do not affect the patient's daily activities
			2: Moderate problem: May include a problem with sleep that exceeds 1, but remains a relatively minor problem (< 50%)
			3: Severe problem: May include a major problem (≧ 50%) with sleep
			Complete problem: May include a complete problem with sleep, such as being incapable of sleeping, or a complete day–night reversal every day
b152	Emotional functions	Extent and frequency of the problem, such as loss of emotional control or lack of emotional expression	0: No problem
			1: Mild problem: May include problems with emotions that do not affect the patient's daily activities
			2: Moderate problem: May include problems with emotions that exceed 1, but remains relatively minor (< 50%)
			3: Severe problem: May include a major problem (≧ 50%) with emotions
			4: Complete problem: May include a complete problem with emotions, such as complete loss of control of emotions, or being incapable of expressing emotions at any time
b280	Sensation of pain	Extent, frequency, and number of sites with pain	0: No problem
			1: Mild problem: May include problems with sensations of pain, but does not affect the patient's daily activities
			2: Moderate problem: May include a problem with sensations of pain that exceeds 1, but remains a relatively minor problem (< 50%)
			3: Severe problem: May include a major problem (≧ 50%) with sensations of pain
			4: Complete problem: May include a complete problem with sensations of pain, such as continuous, intolerable pain
b455	Exercise tolerance functions	Extent and frequency of the problem, such as decline in respiratory and cardiovascular capacity that is required to perform daily activities	0: No problem
			1: Mild problem: May include problems with exercise tolerance that do not affect the patient's daily activities
			2: Moderate problem: May include a problem with exercise tolerance that exceeds Level 1, but remains a relatively minor problem (< 50%)
			3: Severe problem: May include a major problem (≧ 50%) with exercise tolerance
			4: Complete problem: May include a complete problem with exercise tolerance, such as being incapable of bearing any single activity of daily living at any time due to cardiorespiratory problems
b620	Urination functions	Extent and frequency of the problem, such as difficulty urinating or urinary incontinence	0: No problem
			1: Mild problem: May include problems with urination that do not affect the patient's daily activities
			2: Moderate problem: May include a problem with urination that exceeds 1, but remains a relatively minor problem (< 50%)
			3: Severe problem: May include a major problem (≧ 50%) with urination
			4: Complete problem: May include a complete problem with urination, such as complete urinary retention or continuous incontinence at any time
b640	Sexual functions	Extent and frequency of the problem, such as loss of sexual desire and/or physical ability to engage in sexual activity	0: No problem
			1: Mild problem: May include problems with sexual functions that do not affect the patient's daily activities
			2: Moderate problem: May include a problem with sexual functions that exceeds 1, but remains a relatively minor problem (< 50%)
			3: Severe problem: May include a major problem (≧ 50%) with sexual functions
			4: Complete problem: May include a complete problem with sexual functions, such as a complete loss of sexual desire and/or physical ability to engage in sexual activity at any time
b710	Mobility of joint functions	Extent of the problem, such as joint contracture, or limitations in range of motion and percentage of joints with mobility problems	0: No problem
			1: Mild problem: May include problems with joint mobility functions that do not affect the patient's daily activities
			2: Moderate problem: May include problems with joint mobility that exceed 1, but remains a relatively minor problem (< 50%)
			3: Severe problem: May include a major problem (≧ 50%) with joint mobility
			4: Complete problem: May include a complete problem with joint mobility, such as complete joint contracture in all of the major joints
b730	Muscle power functions	Extent of the problem, and percentage of joints with muscle power problems	0: No problem
			1: Mild problem: May include problems with muscle power that do not affect the patient's daily activities
			2: Moderate problem: May include a problem with muscle power functions that exceeds 1, but remains a relatively minor problem (< 50%)
			3: Severe problem: May include a major problem (≧ 50%) with muscle power
			4: Complete problem: May include a complete problem with muscle power, such as a complete loss of muscle power in all of the major muscles

### Modification of the guide—stage 3: second interrater reliability study

A total of 123 patients who underwent rehabilitation from April to June 2020 at Fujita Health University Hospital participated in this study. Among these individuals (78 males and 45 females), 93 had neurological disease, 17 had musculoskeletal disease, 9 had cardiopulmonary disease, and 4 had various other health issues. The mean age of the patients was 69.1 ± 15.1 years. The results of the inter-rater reliability study by four raters (two physical therapists and two occupational therapists) using the final version of the rating reference guide are shown in Table 4. The weighted kappa coefficient was 0.53–0.78, indicating that all categories had moderate to substantial interrater reliability. A high rate of missing data (41.5%) was observed for “b640 sexual functions,” as was seen in the first interrater reliability study. Relative improvements in the weighted kappa coefficients were observed in seven out of the nine categories, except “b620 urination functions” and “b640 sexual functions” (range, -34.4% to 168.8%, median 13.0%).

**Table 4:** Interrater reliability of the rating using the second version of the rating reference guide

	Categories	Weighted κ	95%CI	Relative improvement	Missing values
b130	Energy and drive functions	0.78	0.69–0.87	39.8%	0
b134	Sleep functions	0.65	0.49–0.87	4.2%	0
b152	Emotional functions	0.66	0.54–0.78	168.8%	0
b280	Sensation of pain	0.71	0.62–0.81	60.7%	1 (0.8%)
b455	Exercise tolerance functions	0.60	0.50–0.70	9.1%	0
b620	Urination functions	0.72	0.62–0.81	-22.4%	2 (1.6%)
b640	Sexual functions	0.53	0.32–0.73	-34.4%	51 (41.5%)
b710	Mobility of joint functions	0.65	0.55–0.75	13.0%	0
b730	Muscle power functions	0.74	0.66–0.82	13.4%	0

*95%CI* 95% confidence interval

## Discussion

In the current study, a rating reference guide for the nine body function categories of the ICF Generic-30 Set was developed using a predefined process that involved clinicians’ ratings, cognitive interviews, a field test and reviews by multidisciplinary panels, and interrater reliability studies on the first field-tested version and final (post-field test) version of the guide. The first version of the guide outlined which aspects to rate, gave an example of what should be considered a complete problem, and provided a rating scale of 0–4 without descriptions of the response options. The interrater reliability of the first version revealed low agreement of the ratings among clinicians in several categories. The guide was subsequently modified to produce a final version, which was tested again for interrater reliability. The results of this second interrater reliability study showed moderate to excellent interrater reliability for all categories, indicating an improvement in the guide, from the standpoint of interrater reliability.

The improved reliability of the rating enhances the usability of ICF for clinical and statistical purposes. The results of the clinical measures are anticipated to be consistent across raters and over time. The results can be utilized for the objective clinical assessment of functioning. Information regarding a patient's functioning can then be communicated among various clinicians. The results can also be used for statistical purposes, to evaluate the results of an intervention, or to compare the results across various institutions or regions. For this purpose, it is important to ensure that the evaluation is conducted on common ground. The development of a concrete reference guide with substantial reliability is expected to lay the foundation for the use of ICF for clinical and statistical purposes. This will also contribute to the further implementation of ICF.

Several challenges arose in developing the rating reference guide, one of which was deciding which aspects of the body function problem to focus on when rating. The first version of the rating reference guide addressed this problem by defining the frequency and extent of the problem as aspects of focus. Defining specific aspects to focus on had a positive effect on rating reliability. Indeed, the overall weighted kappa values for the categories were higher in the current study than in previous studies [12, 25]. However, for several items, the weighted kappa coefficients showed lower reliability compared to the good to excellent interrater reliability of the body function-related items in the clinical scales used in previous studies [13, 26, 27]. Moreover, a common point raised by the raters who participated in the first interrater reliability study was the difficulty in deciding between ratings 1 (mild problem) and 2 (moderate problem), apparently due to the lack of a clear explanation of the differences between them. Difficulty in rating functioning using ICF qualifiers, as reflected in the interrater reliability, was addressed in a previous study. Uhlig and colleagues showed that the low interrater reliability of ICF qualifier-based ratings could be improved by collapsing the response options, combining ratings 1 and 2 into a single response option [12]. We used a different approach to address this problem, and added explanations to frame the response options based on the feedback the raters gave during the field test and the review of the notes from the cognitive interviews. The effect on daily activities was mentioned as the difference between ratings 1 and 2; this may prove controversial, since it is important to assess the ICF components of body functions, activities, and participation separately. According to the ICF [1], ICF categories and domains are mutually exclusive. More importantly, rating ICF components independently facilitates the examination of their subsequent relationships. Nevertheless, the impact of body function impairments on daily activities is an important aspect in assessing the overall functioning of patients, and the extent to which the impairment of body functions affects other aspects of functioning is still the focus of rating respective body functions. This reflects the interrelationship between body functions, activities, participation, and contextual factors, as shown in the biopsychosocial model of the ICF [1].

The modifications to the rating reference guide also included changes related to “b152 emotional functions” In the first version of the guide, b152 focused only on losing the control of emotions. However, in rehabilitation clinics, the lack of emotional expression is another frequently observed problem in emotional functions. The lack of emotional expression is a functioning manifestation of depression that is common in many rehabilitation patients [28, 29]. Given this, the modification of the rating reference guide to include lack of emotional expression is justified.

Also revised was the guidance on evaluating “b280 sensation of pain,” that is, adding the number of pain sites as a factor to be considered in the rating based on the interview with the raters in the field test. Previous studies have shown that the number of pain sites is a potential modifier of pain severity and affects the health-related quality of life of patients [30, 31].

After the aforementioned modifications were implemented, the final rating reference guide underwent a second interrater reliability test. The results showed that the weighted kappa coefficients (seven out of nine categories) were improved compared to the first study. Consequently, seven out of nine categories were found to have a weighted kappa coefficient of 0.61 or higher. This indicated substantial reliability for these items. In addition, the lower limit of the confidence interval exceeded 0.61 in four of the nine categories. This reinforces the strength of the results in these categories. Although the weighted kappa coefficients of “b455 exercise tolerance functions” and “b640 sexual functions” were slightly lower than the other categories, the upper limit of the confidence interval extended into the substantial reliability range. Overall, the results of the second study on interrater reliability were comparable to the results of other widely used clinical scales [13, 26, 27], and supports the feasibility of the use of body function categories of the ICF Generic-30 Set in real clinics using the final version of the rating reference guide. The weighted kappa coefficient of the "b640 sexual functions" was high in the first study but diminished in the second study. This may be related to the small sample size (*n* = 19) in the first study that comes from the extremely high number of missing values (68.3%). The rate of missing data in b640 remained high in the second study. This may have been due to the considerable number of elderly participants for whom sexual functions may have been less relevant or possibly also due to the decision by rating clinicians to avoid asking patients about sexual issues. A study of patients with higher requirements for sexual function may contribute to further refinement of the reference guide for "b640 sexual functions".

### Practical implications

International efforts have been made to develop ICF-based clinical tools. The development of such tools includes the development of ICF sets and corresponding simple and intuitive descriptions of the ICF categories contained in such sets [3, 10, 11, 18, 19]. In addition, studies using such ICF sets with simple descriptions have also been conducted [32–34]. The majority of these studies used an intuitive rating scale, whereby "intuitive" means that there is no specific rating guide provided. For example, a project in China with a large sample used the ICF Generic-7 Set with an intuitive rating scale of 0 to 10 [33, 35]. The advantage of an intuitive rating system is that it does not require a complicated process to define each response option. Although we were aware of this approach, we chose to develop a rating reference guide route and specifically to develop a scale system with descriptions of each response option. For one, this approach is similar to that in most clinical scales, and although developing response option descriptions is resource-intensive, this approach has a clear advantage in that clinicians can better understand what each rating in each category means. Clinicians need to understand what they are rating to accurately rate/measure patient functioning. This is especially true considering the feedback from the clinicians in the present study—that it would be better to focus on specific aspects, such as frequency, extent of problem, and influence on daily activity, when rating body function categories. Creating a standard rating guide would not only make rating for clinicians easier, but also help to ensure reliable measurement of patient functioning.

The development of the reference guide (in combination with the guide described by Mukaino et al. which was for activity and participation categories) [14] has resulted in the completion of the reference guide for all items in the ICF Generic-30 set. This development will support the clinical use of the ICF Generic-30 set and foster its clinical implementation. Furthermore, this study proposes a basic structure for a reference guide for the body function categories of the ICF which can be easily extended to other ICF body function categories in the future.

In clinical practice, implementation of ICF may substantially help clinicians to broaden their perspectives regarding patients’ functioning. In rehabilitation practice, the assessment of functioning has primarily focused on activities of daily living (ADL), which describes the activities necessary for independent daily living [36–38]. However, previous studies have also identified other domains of functioning that are important to an individual’s health beyond the concept of ADL [9, 10]. Establishing a reliable and comprehensive functioning assessment system based on ICF contributes to accurate and comprehensive assessment of patients’ level of functioning, increasing the comparability of functioning information and possibly facilitating its use in statistics, which is considered an important role of the ICF.

### Limitations

This study has several limitations. First, the raters and patients included in the first and second interrater reliability studies were different. Thus, the improvement of the weighted kappa scores might be influenced by difference in patient sample and raters. Even so, the use of multiple pairs of raters assessing a statistically sufficient number of patients in the second study would have reduced the risk of bias due to the different sample and raters. Second, the raters in the first interrater reliability study involved individuals who participated in the development process of the rating reference guide. Their knowledge about the rating guide might have affected the results of the first inter-rater reliability study. However, the possible influence would appear to inflate the kappa statistics in the first study and not negate the improvement in the inter-rater reliability in the second study.

Third, the raters who participated in this study were experienced rehabilitation clinicians. Since previous studies have shown that clinical experience could influence interrater reliability [39, 40], the reliability may be lower with less experienced clinicians. Further investigations are necessary to determine whether interrater reliability can be achieved with less experienced rehabilitation clinicians or other allied health professionals, such as nurses, who are less familiar with functional evaluation. If the interrater reliability is affected by the experience in functional evaluation, the development of an education system for the raters and investigation into its effectiveness would be warranted. Another shortcoming of the current study was the characteristics of the patients who participated, that is, a considerable number of the participants were elderly, predominately (89.4%) patients with stroke and orthopedic disease. Nevertheless, since patients with neurological and orthopedic diseases comprise the majority of rehabilitation patients, the present results support the potential use of the rating reference guide in rating rehabilitation patients. Further investigation with a more diverse sample would underscore the generalizability of the findings.

## Conclusion

A rating reference guide for body function categories of the ICF Generic-30 Set was successfully developed, and sufficient levels of interrater reliability were achieved after modifications. This guide is expected to support clinicians in the use of ICF in clinical rehabilitation practice.

## Data Availability

The datasets used and/or analyzed during the current study are available from the corresponding author upon reasonable request.

## Abbreviations

ICF: International Classification of Functioning, Disability, and Health
WHO: World Health Organization
